# Late Diagnosis of Swyer Syndrome in a Patient with Bilateral Germ Cell Tumor Treated with a Contraceptive Due to Primary Amenorrhea

**DOI:** 10.3390/ijerph20032139

**Published:** 2023-01-24

**Authors:** Elżbieta Sowińska-Przepiera, Mariola Krzyścin, Adam Przepiera, Agnieszka Brodowska, Ewelina Malanowska, Mateusz Kozłowski, Aneta Cymbaluk-Płoska

**Affiliations:** 1 Department of Endocrinology, Metabolic and Internal Diseases, Pomeranian Medical University in Szczecin, Unii Lubelskiej 1, 71-252 Szczecin, Poland; 2 Pediatric, Adolescent Gynecology Clinic, Department of Gynecology, Endocrinology and Gynecological Oncology, Pomeranian Medical University in Szczecin, Unii Lubelskiej 1, 71-252 Szczecin, Poland; 3 Department of Urology and Urologic Oncology, Pomeranian Medical University in Szczecin, Al. Powstańców Wielkopolskich 72, 70-111 Szczecin, Poland; 4 Department of Gynecology, Endocrinology and Gynecological Oncology, Pomeranian Medical University in Szczecin, Unii Lubelskiej 1, 71-252 Szczecin, Poland; 5 Department of Reconstructive Surgery and Gynecological Oncology, Pomeranian Medical University in Szczecin, Al. Powstańców Wielkopolskich 72, 70-111 Szczecin, Poland

**Keywords:** Swyer syndrome, DSD, disorders of sex development, amenorrhea, germ cell tumor, dysgerminoma, dysgenetic gonad, ovary

## Abstract

Swyer syndrome is a special form of DSD (disorders of sex development), so-called pure gonadal dysgenesis with a karyotype 46, XY and a female phenotype. One of the most important problems in patients with DSD is the risk of gonadal tumors. We present a case of a 26-year-old patient with Swyer syndrome. The patient had primary amenorrhea and no puberty characteristics. In ultrasound imaging in the vicinity of the uterus, there were two homogeneous structures. A genetic diagnosis was also performed, which showed karyotype 46, XY. The patient underwent a bilateral gonadectomy. Histopathological examination revealed the presence of dysgerminoma in both dysgenetic gonads. The follow-up of five years now did not show any changes suspected of invasion. We concluded that the primary amenorrhea, along with the absence of development of sexual characteristics, should prompt an expanded diagnosis for disorders of sex development. Gonadal dysgerminoma should be suspected even in the absence of tumor features on ultrasound and blood laboratory tests. Early prophylactic gonadectomy could protect patients from developing tumors in dysgenetic gonads.

## 1. Introduction

Disorders of sex development (DSD) are rare, associated with a frequency of 1:1000 births [1]. Many genes are located on the Y or X chromosome, and about 200 autosomal genes are involved in the process of sex differentiation. Mutations in one of these genes already cause disturbances in the differentiation of individual sex traits resulting in malformations, puberty and fertility problems [2]. Swyer syndrome is a special form of DSD, so-called pure gonadal dysgenesis, in which there is a gender reversal because these individuals have a male karyotype 46XY and a female phenotype [3]. In this syndrome, sex determination disorders are caused by structural mutations or aberrations in the SRY gene region on the Y chromosome. The protein encoded by SRY is a regulatory transcription factor that disables the activity of the WTN4 DAX1 gene that blocks the processes leading to testicular development. In the situation where the SRY protein, SRY gene or the entire Y chromosome is missing, along with the presence of dysgenetic gonads, it comes to the development of female sexual characteristics [4]. One of the most important problems in patients with disorders of sex development is the risk of embryotic gonadal tumors. Patients who possess genetic material from the Y chromosome are particularly at high risk [5]. The risk is shown in Table 1.

Diagnosis of Swyer syndrome is based on medical history and physical examination, determination of gonadotropin and sex hormone levels, imaging (ultrasound, MRI), analysis of cytogenetic and molecular tests and histological assessment of gonads [3]. The interview should focus on genetic diseases and gonadal cancers in the family, assessment of intellectual development, past and present diseases and whether there are symptoms of endocrine diseases. In addition, it is important to evaluate the rate of growth and weight gain, look for dysmorphic traits, assess the formation of secondary and tertiary sexual characteristics, the presence and quality of menstrual bleeding and possible fertility problems. The clinical manifestation usually does not occur during childhood. Swyer syndrome usually reveals itself in adolescence as delayed puberty and primary amenorrhea [7].

Our paper presents a case of a patient with primary amenorrhea, in whom no investigation was performed, and a late diagnosis of gonadal dysgenesis with karyotype 46XY resulted in the development of bilateral gonadal neoplasm. The aim of the study is to emphasize the importance of early implementation of diagnostic procedures in the case of primary amenorrhea in patients with Y chromosome-derived genetic material.

## 2. Case Report

**Figure 1:**
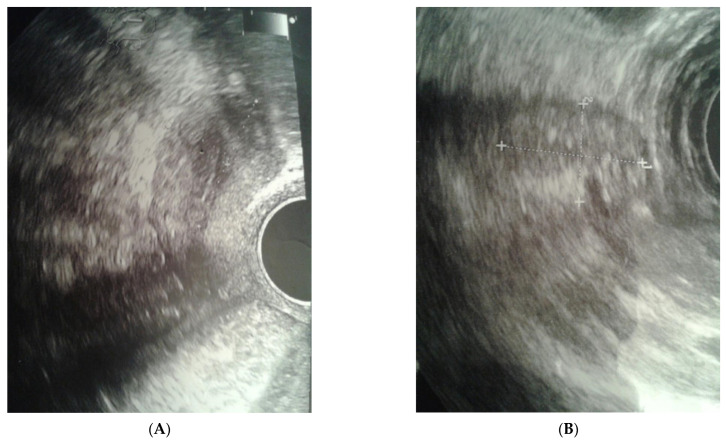
Ultrasound before surgery: (**A**) uterus, (**B**) gonad left.

The clinical case presented, aged 26, came to the Gynecological Endocrinology Clinic due to reproductive plans and a lack of menstruation occurring for several years. From the age of 16 to 19, she was treated with oral contraception due to primary amenorrhea. During treatment, the bleeding occurred regularly. Based on the interview at that time, no diagnostics but a gynecological examination was performed. For unspecified reasons and a lack of support in the family, the female stopped treatment. The woman was 168 cm in height and 59 kg in weight. Physical examination revealed no puberty characteristics: development of mammary glands: scanty glandular tissue, pale nipple envelopes and papillae (Thelarche 2/3); scanty pubic hair (Pubarche 2); axillary hair (Axillarche-2) in addition to a narrow, childlike pelvis. The gynecological and imaging examinations revealed the presence of a hypoplastic uterus (28.0 mm × 23.1 mm) with a linear endometrium and normal cervix. In the vicinity of the uterus, there were two homogeneous structures (27 × 18 × 12 mm and 15 × 13 × 8 mm) that may correspond to hypoplastic ovaries. No pelvic abnormalities were found (Figure 1).

Gonadotropin concentrations were determined, which were: FSH—56.7 mIU/mL, LH—19.8 mIU/mL, sex steroid concentrations: E2 < 5 pg/mL, T—0.348 ng/mL; in addition, the concentrations: 17-OHP—1.03 ng/mL, TSH—3.7 mIU/mL, fT4—1.03 ng/dL, ACTH—28.6 pg/mL, cortisol—16.9 ug/dL, tumor markers: LDH—118 U/L, β-HCG—0.753 mIU/mL, AFP—3.88 IU/mL. Detailed hormone profiles and tumor markers are shown in Table 2.

On the basis of the overall clinical picture and the results of previous studies, hypergonadotropic hypogonadism was found, and the patient was referred for cytogenetic testing. Since then, the female underwent intensive psychological care both in the form of individual and group therapy.

**Figure 2:**
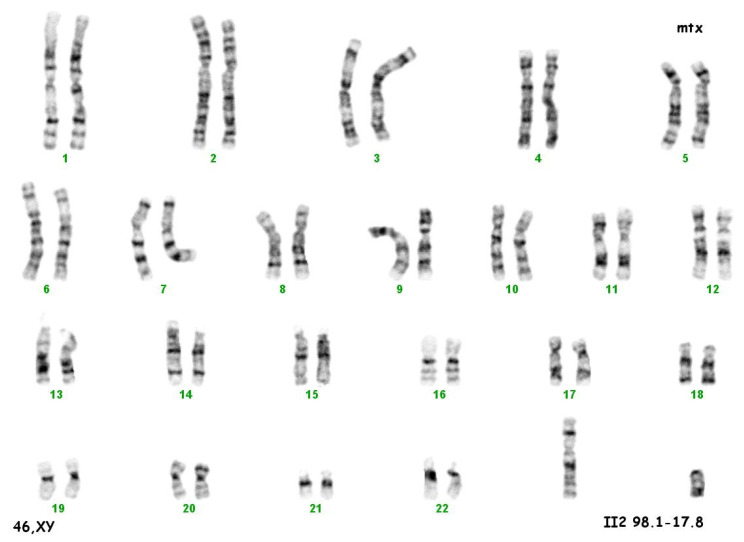
Karyotype 46 XY.

The culture of peripheral blood lymphocytes revealed the presence of the SRY gene sequence and other genes within the Y chromosome (ZFY, ZFX, AZFa—sY84, sY86, AZFb—sY127, sY134, AZFc—sY254, sY255) (Figure 2).

The final diagnosis was made: DSD with karyotype 46, XY.

**Figure 3:**
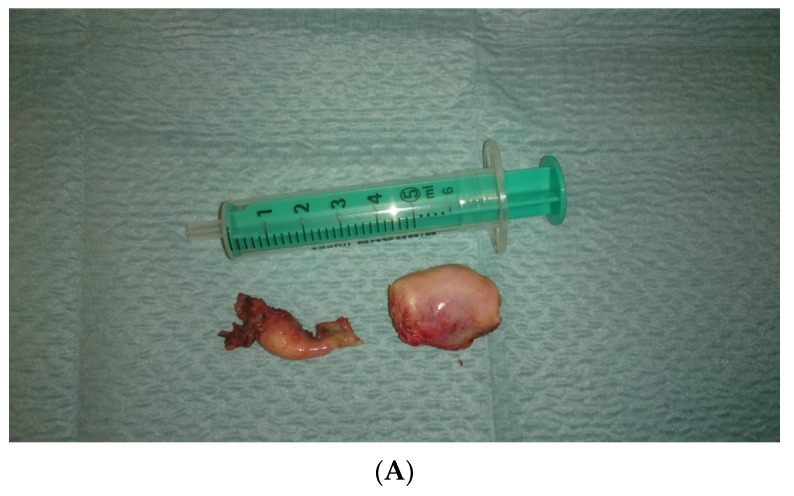

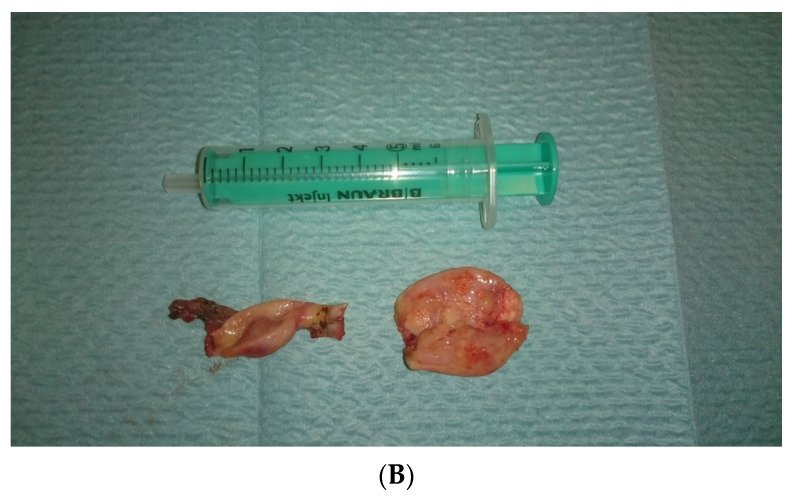
(**A**,**B**). Right and left gonad after surgery.

**Figure 4:**
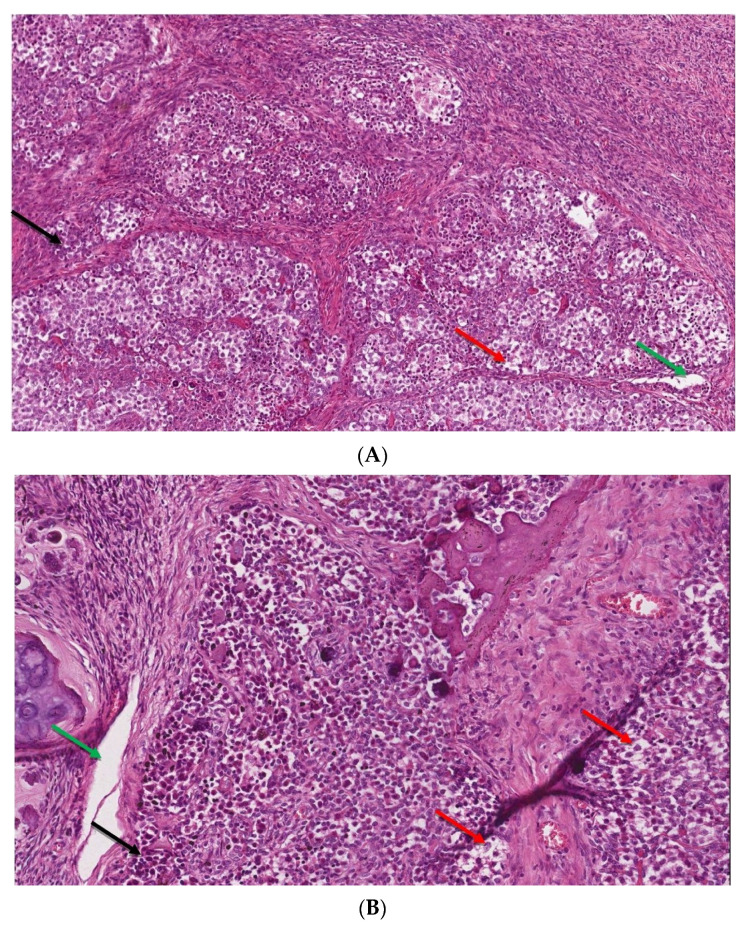
(**A**,**B**). The microscopic image of dysgerminoma: (**A**) monomorphic, polygonal in shape tumor cells, with well-visible cell borders, clear or lightly eosinophilic cytoplasm, large vesicular nuclei in the center (red arrow). The tumor cells are arranged in diffuse nests separated by delicate fibrous septae containing inflammatory cells (black arrow) (HE, ob. 10×); (**B**) details of the described area, visible pseudoglandular spaces or poorly-formed tubules due to discohesion among tumor cells (green arrow) (HE, ob. 20×).

In view of the normal macroscopic image of the gonads, the laparoscopic salpingo-ooferectomie was performed (Figure 3). Histopathological examination revealed the presence of dysgerminoma in both dysgenetic gonads (Figure 4).

Due to the malignant nature of the tumor and the performance of a conservative procedure, the decision of a close further observation decision was made. It involved the determination of tumor markers, alkaline phosphatase concentration, and CT imaging of the abdominal cavity, pelvis and chest CT. Additionally, an adjuvant hormonal estrogen-progestagen therapy was started to obtain regular uterine bleeding and strengthen the sense of sex. 

The follow-up of five years now did not show any changes suspected of invasion. Tumor markers levels after surgery were within normal limits; LDH—135 U/L, β-HCG—0.815 mIU/mL, AFP—2.88 IU/mL, Ca-125—19.01 U/mL.

## 3. Discussion

The most important threats for women resulting from chronic amenorrhea depend on the period of life in which the disorder occurs and include delayed puberty, infertility and distant consequences: failure to achieve peak bone mass or its rapid loss. Primary amenorrhea is defined as a lack of menstruation in a 13-year-old girl who has underdevelopment of secondary sexual characteristics or a lack of menstruation in a properly developed 15-year-old girl [8]. Based on the etiology, amenorrhea can be classified as: outflow tract abnormalities, primary ovarian insufficiency, hypothalamic or pituitary disorders, other endocrine gland abnormality such as thyroid or adrenal gland disorders and other [9]. The most frequent causes of amenorrhea are ovarian failure (43%), followed by Müllerian agenesis (15%) and constitutional delay of puberty (14%) [10]. Detailed diagnostics of amenorrhea should begin with a taking history and thorough physical examination. The interview should include elements such as eating disorders, excessive physical exertion, personality traits (perfectionism and the need for social acceptance, high ambitions and high demands), stressors, mood disorders, weight changes, amount of sleep and the intake of addictive substances. A detailed family history should also be collected. In any case of amenorrhea, including primary amenorrhea, pregnancy should be excluded as it is the most common physiological cause of this phenomenon. In physical examination, special attention should be paid to the development of secondary sexual characteristics in addition to body weight, height, hyperandrogenism, galactorrhoea, thyroid enlargement or clitoral hypertrophy. Afterwards, an ultrasound or MRI of the pelvis should be performed, allowing the determination of the presence of the uterus and assessment of its structure. Abnormal development of secondary sexual characteristics should suggest a suspicion of hormonal disorders. Assessment of the levels of tropic hormones (FSH, LH), estradiol and progesterone is always needed; the testing of other hormones adapts to clinical symptoms. Ambiguous genital organs and uterine detection or finding abnormal structure in imaging is an indication to determine the karyotype that allows the final diagnosis of androgen insensitivity syndrome (46, XY), Swyer syndrome (46, XY) or MRKH syndrome (46, XX) [9]. Actually, the genetic causes of DSD are highly heterogeneous, thereby posing a diagnostic challenge. Baxter et al. reported whole exome sequencing followed by an analysis of selected DSD genes as having a diagnostic yield of 22.5% in patients with 46, XY DSD [11]. A more recent study revealed a relatively higher yield by a targeted panel sequencing approach, in which a genetic diagnosis was made in 38.1% [12]. Considering the diagnostic potential of a high-throughput approach, next-generation sequencing (NGS) panels for DSD could be used in practice to uncover the underlying genetic causes of primary amenorrhea [13].

In addition to gonadectomy, which is used in high-risk patients, hormone replacement therapy (HRP) should also be implemented [14]. For primary ovarian insufficiency, HRP consists of 100 mcg of transdermal estradiol per day or 0.625 mg of oral conjugated estrogens per day and the addition of 200 mg of micronized oral progesterone per day for 12 days each month. Treatment with estrogens administered in non-oral form, preferably percutaneous, is proposed because of the lower risk of thromboembolic complications [9]. In people with the female phenotype, the presence of the Y chromosome or gene sequence derived from the Y chromosome in dysgenetic gonads significantly increases the risk of developing germ cell tumors [15]. So far, a number of markers associated with the development of GCT (germ cell tumor) have been identified, the most important of which are TSPY (testis-specific protein-Y) and OCT3/4 (octamer binding transcription factor 3/4) [16]. However, each time when diagnosed 46, XY dysgenesis, there are indications for the prophylactic removal of gonads immediately upon diagnosis [15]. Persistent expression of OCT3/4, the presence of c-KIT (the stem cell factor receptor) and PLAP (placental-like alkalinephosphatase) antibodies are recognized markers of tumors originating from primary sex cells: carcinoma in situ (CIS), gonadoblastoma (GB) and overt GCT [17]. GB is somehow a precursor of GCT, so early detection and the use of appropriate treatment in preventing cancer deformation is crucial in this matter. Huang et al. studied the frequency of tumor transformation in the gonads of people with DSD. The highest incidence of tumors (23–33%) was observed in people with Swyer syndrome, with a very high percentage of tumor transformation, which reached 62% and was the highest among the known DSDs [18]. 

The Bandala-Jacques et al. study compared optimal and sub-optimal cytoreduction for ovarian dysgerminoma. They concluded that initial residual disease did not significantly affect progression, recurrence, overall survival or disease-free survival. However, it should be noted that cases in stages III and IV were analyzed [19]. In our case described here, gonad removal was performed, as both ultrasound images and blood markers did not suggest the presence of ovarian neoplasm. Only the result of the histopathological examination showed dysgerminoma. Laparoscopic procedures on the gonads, on the other hand, were studied by Wünsch et al. Patients with complete gonadal dysgenesis were found to have highly variable gonadal morphology, demonstrating the presence of neoplasm in some cases [20].

## 4. Conclusions

Based on the case we described, we drew the following conclusions: The primary amenorrhea, along with the absence of development of sexual characteristics, should prompt an expanded diagnosis for disorders of sex development. Gonadal dysgerminoma should be suspected even in the absence of tumor features on ultrasound and blood laboratory tests. Early prophylactic gonadectomy could protect patients from developing tumors in dysgenetic gonads.

## Figures and Tables

**Table 1:** The risk of developing germ cell tumors depending on the main diagnosis (according to LWPES/ESPE)**.** Table based on the work of Hughes et al. [6].

Group of Risk	Malignancy Risk [%]	Disorder	Procedure
High	15–35	gonadal dysgenesis (Y +, intraabdominal gonads)	gonadectomy (during the diagnosis period)
	50	PAIS (testicles outside the scrotum)	
	40–60	Frasier, Denys Drash (Y+)	
Middle	12	Turner (Y+)	gonadectomy (during the diagnosis period)
	28	17ß HSD deficiency	Monitoring
	?	Gonadal dysgenesis (Y +, gonads in the scrotum)	Biopsy during puberty and irradiation ?
	?	PAIS—partial insensitivity to androgens (gonads in the scrotum)	Biopsy during puberty and irradiation ?
Low	3	CAIS—complete insensitivity to androgens	Biopsy during puberty and ?
	2	Ovarian-nuclear DSD	Testis tissue removal ?
	1	Turner (Y-)	None

PAIS—partial androgen insensitivity syndrome; CAIS—complete androgen insensitivity syndrome; ?—unknown occurrence frequency; DSD—Disorders of sex development.

**Table 2:** Hormonal profile and tumor markers.

Parameter	Score	Sr
FSH	56.7 mIU/mL	2.8–11.3
LH	19.8 mIU/mL	1.2–11.6
Estradiol	<5 pg/mL	10–160
Testosteron	0.348 ng/mL	0.006–0.8
ACTH	28.6 pg/mL	7.2–63.3
Cortisol	16.9 µg/dL	6.2–19.4
TSH	3.7 mIU/mL	0.2–4.0
fT4	1.03 ng/dL	0.9–1.7
LDH	118.0 U/L	120–230
beta-hCG	0.753 IU/mL	<10
AFP	3.8 IU/mL	<6
Ca-125	8.21 U/mL	<35

FSH—follicle-stimulating hormone; LH—luteinizing hormone; ACTH—adrenocorticotropic hormone; TSH—thyroid-stimulating hormone; fT4—free thyroxine; LDH—lactic dehydrogenase; AFP—alpha-fetoprotein; beta HCG—human chorionic gonadotropin; Sr—standard range.

## Data Availability

No new data were created or analyzed in this study. Data sharing is not applicable to this article.
